# The preparation and application of a graphene-based hybrid flame retardant containing a long-chain phosphaphenanthrene

**DOI:** 10.1038/s41598-017-09459-9

**Published:** 2017-08-18

**Authors:** Wenhua Chen, Yuansen Liu, Pengju Liu, Changan Xu, Yuan Liu, Qi Wang

**Affiliations:** 1 Polymer Research Institute of Sichuan University, The State Key Laboratory of Polymer Materials Engineering, Chengdu, 610065 China; 2 Engineering Research Centre of Marine Biological Resource Comprehensive Utilization, Third Institute of Oceanography, State Oceanic Administration, Xiamen, 361005 China

## Abstract

A novel hybrid flame retardant combining graphene oxide (GO) with long-chain phosphaphenanthrene was fabricated via surface grafting reaction. Taking advantageous of the double barrier effects, including the physical shield contributed by graphene nanoplates during the initial stage and the chemical char contributed by phosphaphenanthrene during the later stage, greatly decreased the release rate of decomposed volatiles from the resin, as well as minimized the release of oxygen and combustion heat. Hence, such hybrid flame retardant can overcome the shortcomings of early acid catalyzed degradation effects caused by conventional flame retardants containing phosphorus. Satisfactory flame retardance was achieved (UL94 V-0 rating) with only 4% addition of the hybrid flame retardant to the epoxy resin laminate. Due to the long-chain and bulky phosphaphenanthrene groups, the interlayer attractive forces of the modified GO were effectively weakened, thus favoring the exfoliation and dispersion of graphene sheets. As a result, the incorporation of the flame retardant slightly enhanced the mechanical properties of the polymer composites, rather than deteriorating them, as occurs with traditional additive flame retardants. As a potential application for graphene, it is believed that the reported hybrid flame retardant has promising future prospect.

## Introduction

Polymers are widely applied in modern society due to their excellent properties and abundant product forms. However, high flammability is a general shortcoming of these materials. To endow polymers with flame retardance, various flame retardants are introduced into the polymer matrix. As an important halogen-free flame retardant, phosphorus flame retardants (PFRs) have attracted extensive attention for their high efficiency^1^. The working mechanism of most PFRs can be described as catalysis charring during polymer combustion^2^. At high temperature, PFRs decompose into phosphoric acids, which effectively catalyze the transformation of the polymer matrix into graphitized char, and the resultant dense charring layer can cover the material surface to isolate the fire, oxygen and heat^3, 4^. However, the existing PFRs still have some problems. On one hand, almost all additive flame retardants, including PFRs, lead to obvious deterioration of the processability and mechanical properties of polymers due to poor compatibility between materials^5, 6^. On the other hand, the acids released from PFRs have two catalytic actions: they not only catalyze the charring process^7^ (during the high-temperature stage), but also promote polymer decomposition^8^ (during the initial elevated-temperature stage). The latter can accelerate the release of combustible volatiles from the decomposed polymer and actually intensify the flame in the initial stage, which results in a decrease in the flame retarding efficiency of PFRs to an extent.

To further improve the efficiency of PFRs, synergistic systems with other flame retardants (physical blends or chemical combinations), are generally employed. P-N and P-Si are representative synergistic systems that are usually applied. For the former, inert gases from N elements can expand the char to form an intumescent and cellular structure, which can more effectively block the fire^9–11^. For the latter, organic Si is converted to inorganic SiO_2_ networks at high temperature, which incorporate the graphitized char contributed by PFRs, thus greatly improving the strength, stability and barrier properties of the char layer^12–14^. In addition, it has been reported that the incorporation of metal salts with PFRs can also show synergistic effects^15^. The formation of metal phosphate, caused by Lewis acid–base interactions between the metal (acid) and phosphate (base), provides a very effective barrier in the condensed phase^16^. It is clear that the above synergistic systems improve the flame retardance by forming charring layers with higher quality. However, for synergistic technologies, it is difficult to overcome the rapid release of combustible volatiles caused by the catalytic decomposition of PFRs during the initial combustion stage.

Graphene, a two-dimensional carbon material with excellent barrier properties, has been introduced into polymeric materials to enhance their flame retardance, as discussed in previous references^17, 18^. As a physical carbon source, graphene can reduce the heat release and inhibit the transfer of combustible gases during combustion, however, the real flame retardance of polymers, reflected by the vertical flame test rating and limiting oxygen index (LOI), is not notably increased by using graphene alone as a flame retardant. Another challenge is to achieve a good dispersion of graphene throughout the polymer matrix by weakening the attractive van der Waals forces between the graphene sheets. Graphene oxide (GO) obtained using the Hummers’ method contains various oxygen-containing functional groups on the basal planes and edges, which provide an effective approach to realizing the surface decoration of GO^19^. As is well known, decorated GO can be better dispersed in polymers. In addition, as a nano plate-shaped filler that can be well-dispersed in a resin matrix, decorated GO can also enhance the mechanical properties of polymers from a structural point of view^20–22^. Hu^23^ synthesized the functionalized graphene oxide (FRGO) via *in situ* condensation polymerization and applied it to an epoxy resin. A notable reduction in the fire hazard of the nanocomposite was achieved by the addition of FRGO. The effect of graphene nanosheets (GNS) combined with traditional flame retardants was systematically studied by Wang^24^, and different synergistic mechanisms were proposed for different flame retardant systems. In addition, a series of novel graphene-based flame retardants^25, 26^ were also prepared by grafting various substances onto the surface of GO. However, few papers have reported the application of graphene in improving the flame retarding efficiency of PFRs, by taking advantage of the barrier properties of graphene sheets.

In this research, a type of GO decorated by a PFR, 10-dihydro-9-oxa-10-phosphaphenanthrene-10-oxide-g-(2,3-epoxypropoxy) propyltrimethoxysilane (DPP) was prepared via surface grafting. As a novel flame retardant, this material shows the advantages of both GO and PFR, and meanwhile can overcome their respective shortcomings. Making use of the physical carbonaceous shield formed by the GO sheets, this material can prevent the rapid release of combustible gases during the initial combustion stage due to the acid-catalysis degradation effect of PFR, therefore effectively controlling the extension of the early flame. The initial barrier effects contributed by the graphene sheets allow the slower chemical charring behaviour of PFR to occur. The later-produced chemical chars encapsulates the graphene sheets to construct a composite carbonaceous layer with better strength, compactness and stability compared with a single physical or chemical method. As a result, the flame retardant system can maintain good shield effects during the entire process, and for this reason, the system exhibits higher efficiency. Moreover, the long-chain and bulky PFR grafted onto GO weaken the van der Waals forces and expand the space between neighboring GO sheets due to “steric propping-open effects”, which promotes the dispersion and exfoliation of the GO nanosheets. In addition, the extended chains on the graphene sheets easily entangle with the polymer chains and thus enhance the interfacial combination between graphene and the polymer matrix.

## Results

### Structural and properties characterization of DPP-GO

**Figure 1:**
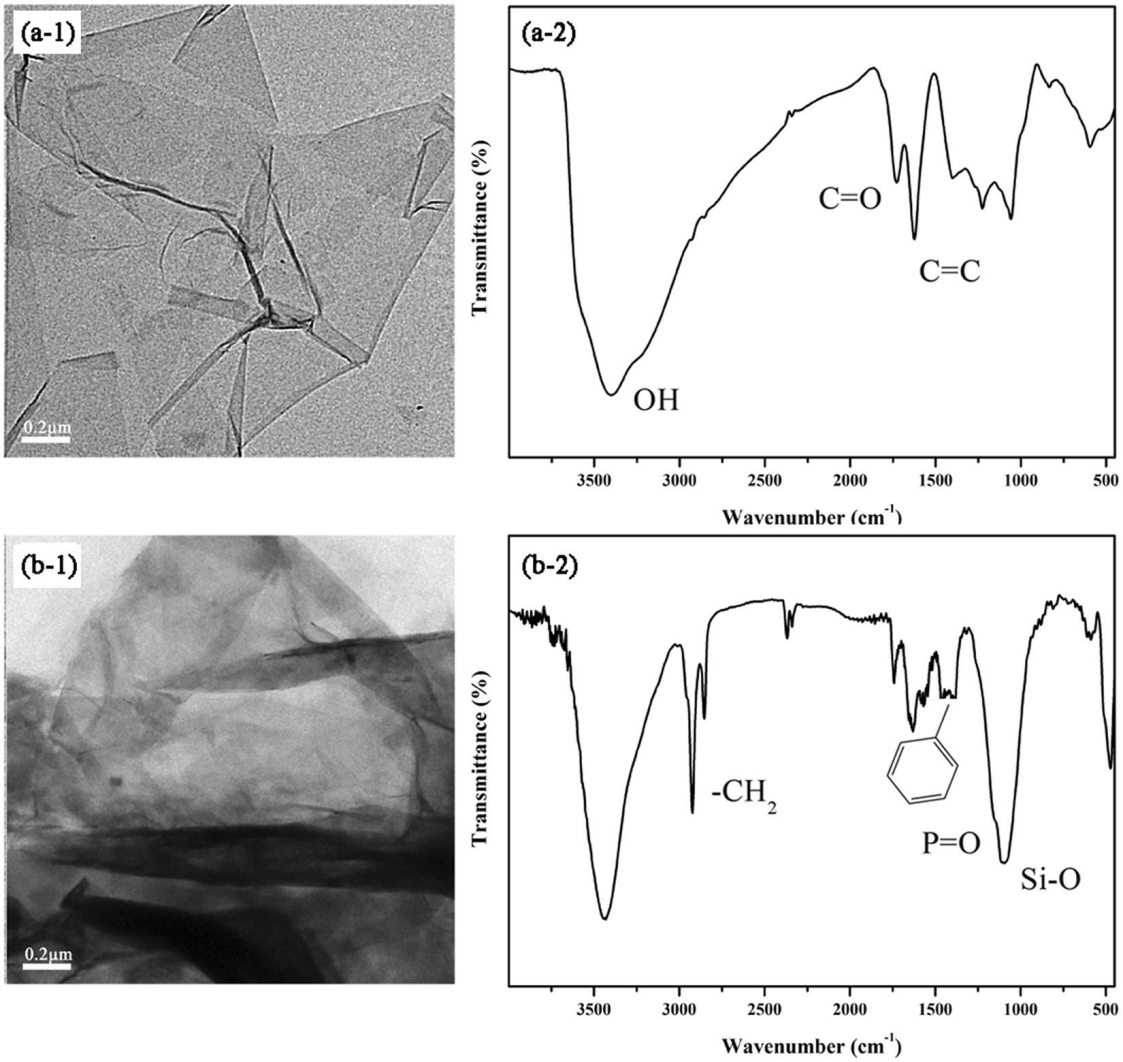
TEM images and FTIR spectra of GO (**a**) and DPP-GO (**b**).

First, the morphologies of GO and the synthesized DPP-GO system were compared by TEM as shown in Fig. 1. The GO sheets exhibited semi-transparent, wrinkled and folded nanoplatelets. In comparison, the edges of DPP-GO became rougher, but the material still maintained good transparency. Their corresponding FTIR spectra were also analyzed. A series of GO characteristic absorption peaks were observed at 3402 cm^−1^(O-H stretching vibration), 1726 cm^−1^ (C = O stretching vibration), 1625 cm^−1^ (C = C stretching vibration), 1410 cm^−1^ (O-H bending vibration), 1223 cm^−1^ (C-O stretching vibration of epoxide) and 1048 cm^−1^ (C-O stretching vibration of alkoxy). In the spectrum of DPP-GO, the peaks at 2850 cm^−1^ and 2919 cm^−1^ were assigned to the symmetric and antisymmetric vibrations of methylene, while the peak at 1210 cm^−1^ was assigned to the Si-O stretching vibration, and the typical absorption peaks between 1400 and 1600 cm^−1^ were assigned to the benzene ring. The appearance of the above characteristic absorption peaks indicated that DPP was successfully grafted onto the GO surface.

**Figure 2:**
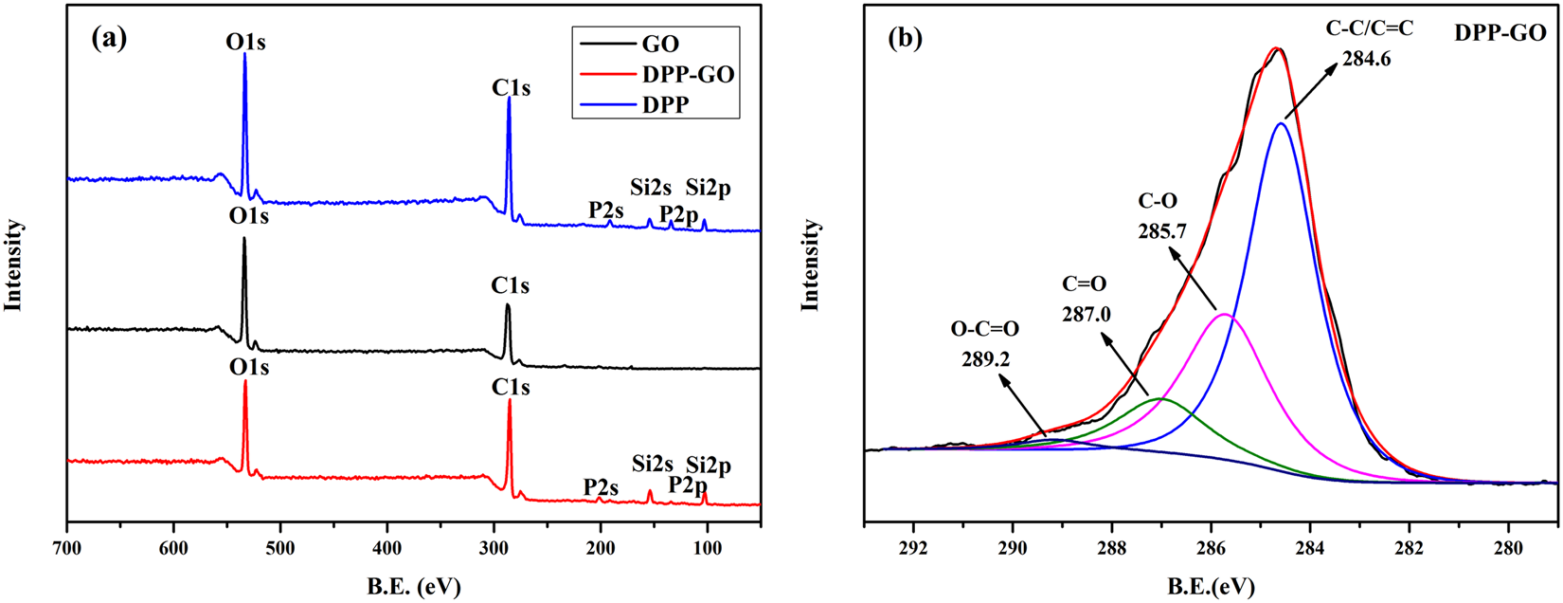
XPS survey spectra of GO, DPP and DPP-GO (**a**), and C1s spectrum of DPP-GO (**b**).

The quantitative analysis of the chemical elemental compositions of DPP-GO was performed by XPS (Fig. 2). It can be seen that only two elements, O and C were detected in the spectrum of GO, while extra peaks attributed to P and Si, were observed in the spectrum of DPP-GO. Moreover, in the high-resolution C1s spectrum of DPP-GO, four absorbance peaks were distinguished: 284.6 eV was attributed to the contribution of C-C and C = C in the GO skeleton, 285.7 and 287 eV were attributed to C-O and C = O, respectively; and 289.2 eV was attributed to O = C-O. The XPS analysis further confirmed the chemical bonding between DPP and GO. In addition, the atom percentages of C, O, Si and P element of DPP-GO were listed in Table 1. Thus, according to the determined P content (1.29 at%) on the surface of DPP-GO, the calculated grafting ratio of DPP on GO was approximately 36 wt%.

**Table 1:** The atom percentages of various elements in DPP-GO.

Sample	C (at%)	O (at%)	Si (at%)	P (at%)
GO	66.42	33.58	—	—
DPP	62.12	29.63	4.51	3.74
DPP-GO	68.24	28.45	2.02	1.29

**Figure 3:**
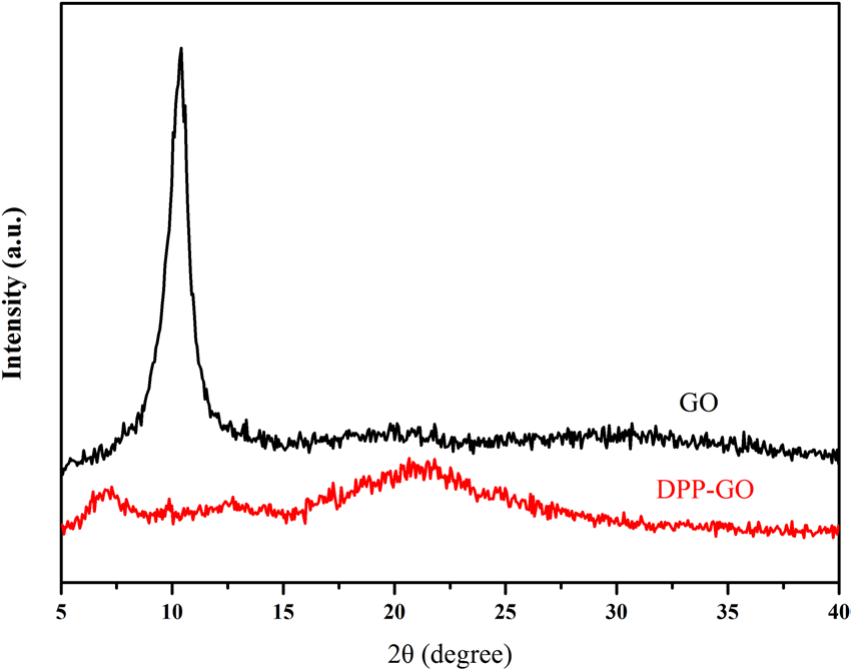
XRD patterns of GO and DPP-GO.

The XRD patterns of GO and DPP-GO are shown in Fig. 3. The typical diffraction peak at 2θ = 10.4° was assigned to GO, indicating an interlayer distance of 0.85 nm. In the DPP-GO spectrum, two distinct peaks at 7.5° and 22° were observed. The former peak (2θ = 7.5°) was shifted to smaller angle than that of GO (2θ = 10.4°), which indicated an increase in the d-spacing from 0.85 nm (GO) to 0.96 nm (DPP-GO) due to the introduction of the long-chain and bulky groups of DPP. Such functionalization enhanced the steric hindrance and favored the separation between graphene sheets. In addition, the obviously weakened intensity of the diffraction peak (2θ = 7.5°) indicated that the grafted DPP also partially damaged the regular stacking of GO.

**Figure 4:**
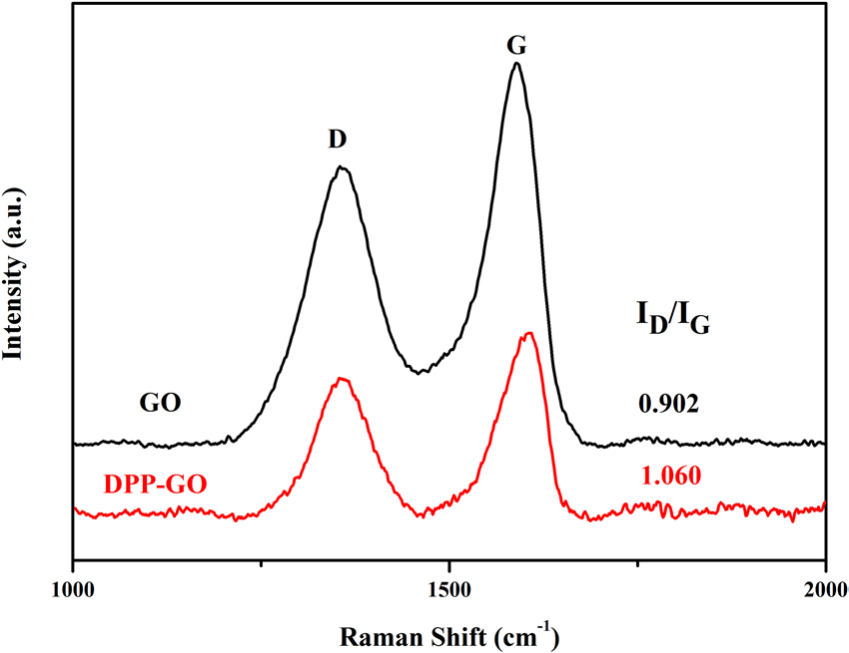
Raman spectra of GO and DPP-GO in the narrow range of 1000–2000 cm^−1^ (D and G bands).

Raman spectroscopy was conducted to further investigate the corrugated structure of GO and DPP-GO as shown in Fig. 4. The in-phase vibration of the sample lattice (G band) at 1570 cm^−1^ and the disorder band (D band) at approximately 1355 cm^−1^ were detected in the spectrum of both GO and DPP-GO^27^. The intensity ratio of the D and G band is a key parameter to evaluate the structure of graphene. The ID/IG ratio was 0.902 and 1.060 for GO and DPP-GO, respectively. The slight increase in the D/G intensity ratio of the latter indicates an increase in amorphous carbon compared to the sp^2^-hybridized graphene due to the introduction of DPP.

**Figure 5:**
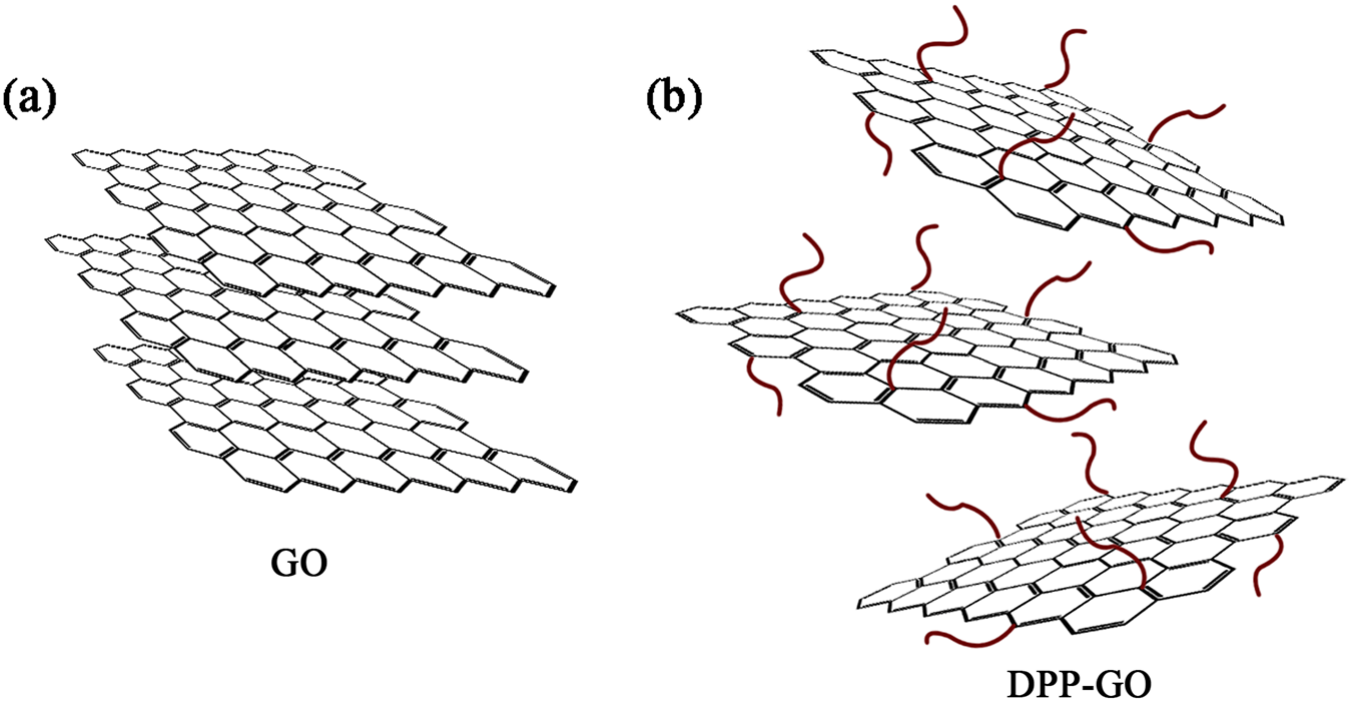
Steric hindrance separates the graphene sheets in DPP-GO.

Both the XRD and Raman analysis confirmed an increase in the interlayer distance and disorder degree for DPP-GO compared to GO. The difference resulted from the long-chain and bulky groups of the former generating remarkable steric hindrance effects, which “props open” the graphene sheets. The mechanism is illustrated in Fig. 5.

**Figure 6:**
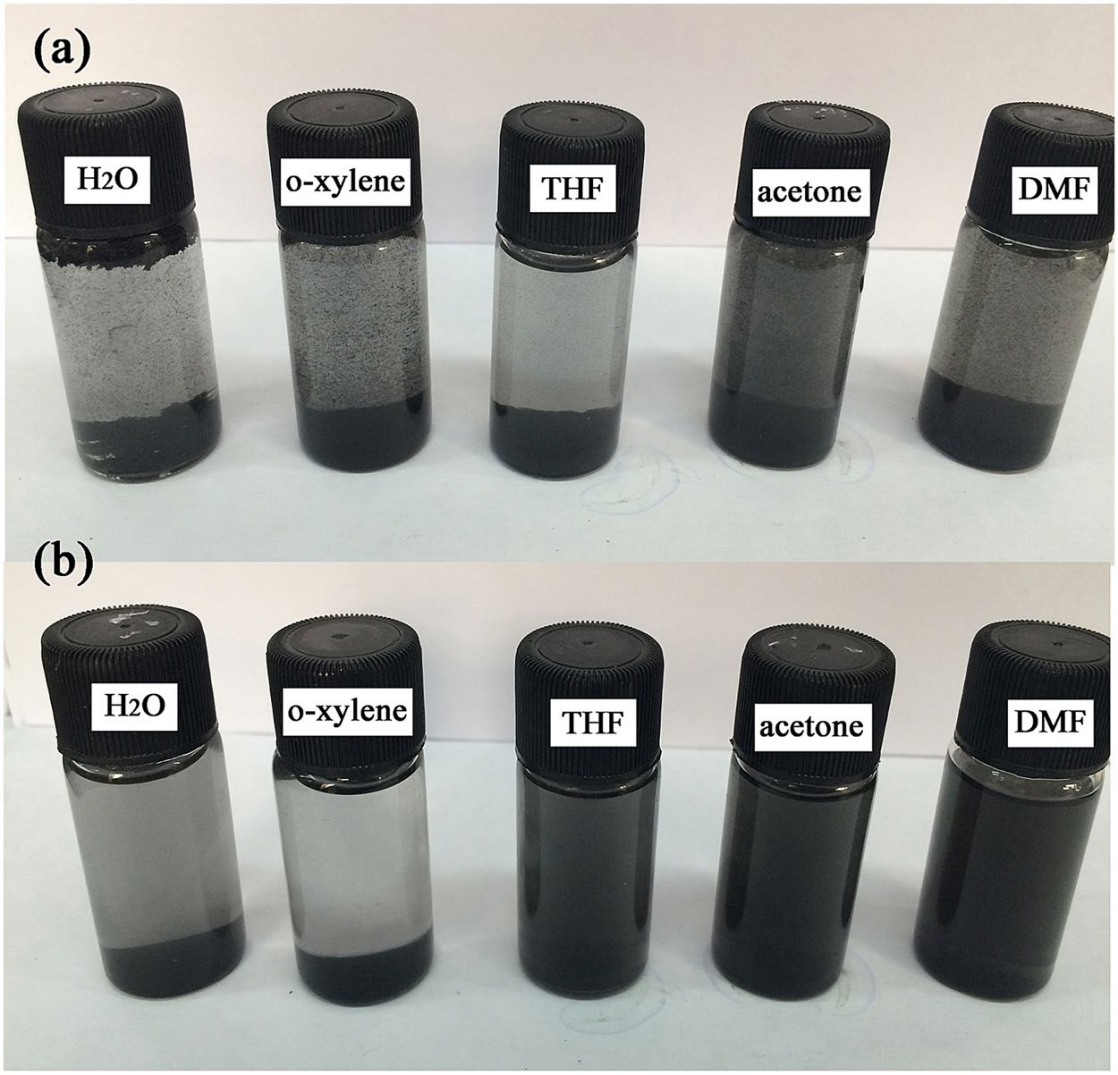
rGO (**a**) and DPP-GO (**b**) dispersed in different solvents.

The dispersion and interface problems are key factors for preparation of the polymer/graphene composites. Functionalization of the graphene surface can generally enhance its dispersion. Here, the dispersion behaviors of reduced graphene oxide (rGO) and DPP-GO in different solvents including water, o-xylene, THF, acetone and DMF, were evaluated. From the photos as shown in Fig. 6, it can be seen that rGO particles were deposited on the bottom in all solvents, showing a very poor dispersion of rGO. In contrast, DPP-GO formed stable colloidal suspensions in THF, acetone and DMF. The results further confirmed that the introduction of DPP was helpful to reduce the compact stacking and improve the dispersion. As acetone and DMF are good solvents for the EP resin, a homogeneous dispersion of DPP-GO in these solvents is advantageous to obtain a high-quality flame retardant EP glue.

**Figure 7:**
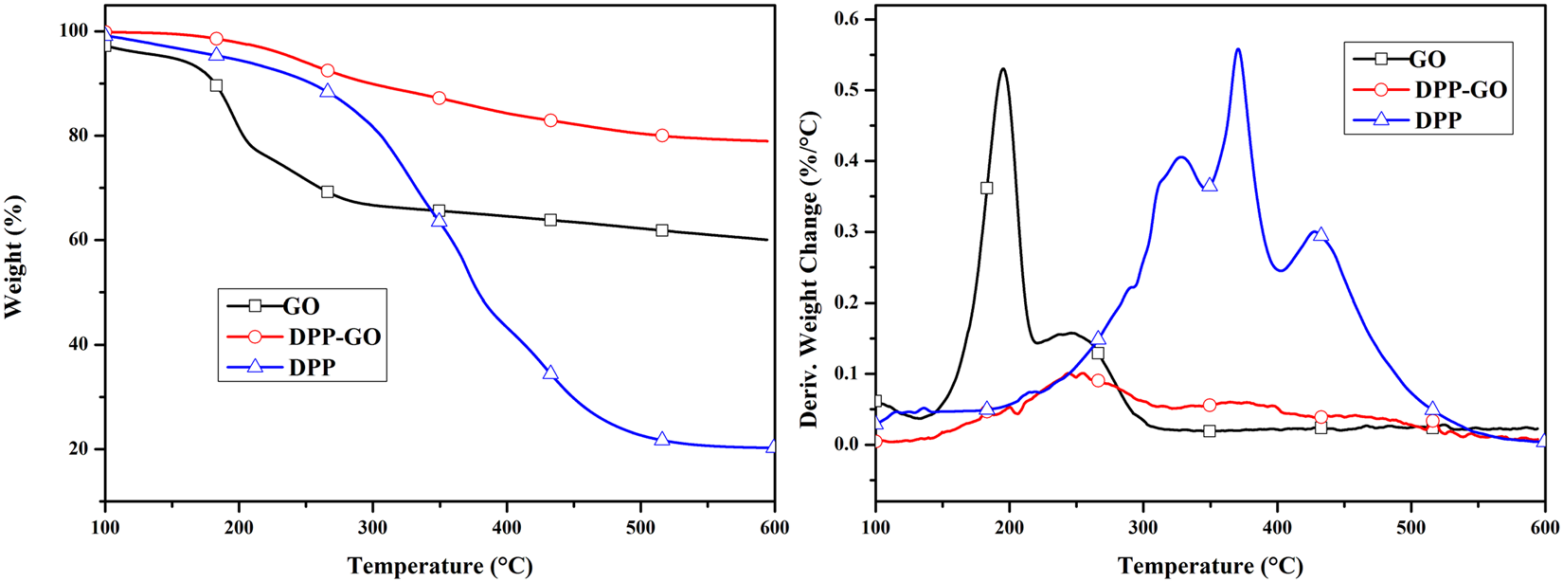
TG and DTG curves of GO, DPP and DPP-GO.

Thermal properties of GO, DPP and DPP-GO were studied using TGA, and their degradation curves are plotted in Fig. 7. A slight drop below 100 °C was recorded in the TG curve of GO which was attributed to the evaporation of residual water in the samples, and the major weight loss peak occurred at around 200 °C due to removal of the various oxygen-containing functional groups including hydroxyl, epoxy and carboxyl groups. Obviously, the poor thermal stability of GO is disadvantageous to its application served as an additive flame retardant. In comparison, as a result of the partial replacement of the oxygen-containing groups with DPP, the initial degradation temperature (at 5 wt% mass loss) of DPP-GO was increased to 215 °C, and its major weight loss peak was increased to 250 °C. The remarkably improved thermal stability of DPP-GO was mainly attributed to the rigid structure of phosphaphenanthrene and the high bond energy of Si-O. Meanwhile, DPP-GO also had a greatly enhanced char yield ratio as high as 80% at 600 °C (only 60% for GO). It is concluded that grafted DPP produced more stable chars at high temperature.

### Flame retardance and mechanism of DPP-GO flame retardant EP composites

**Figure 8:**
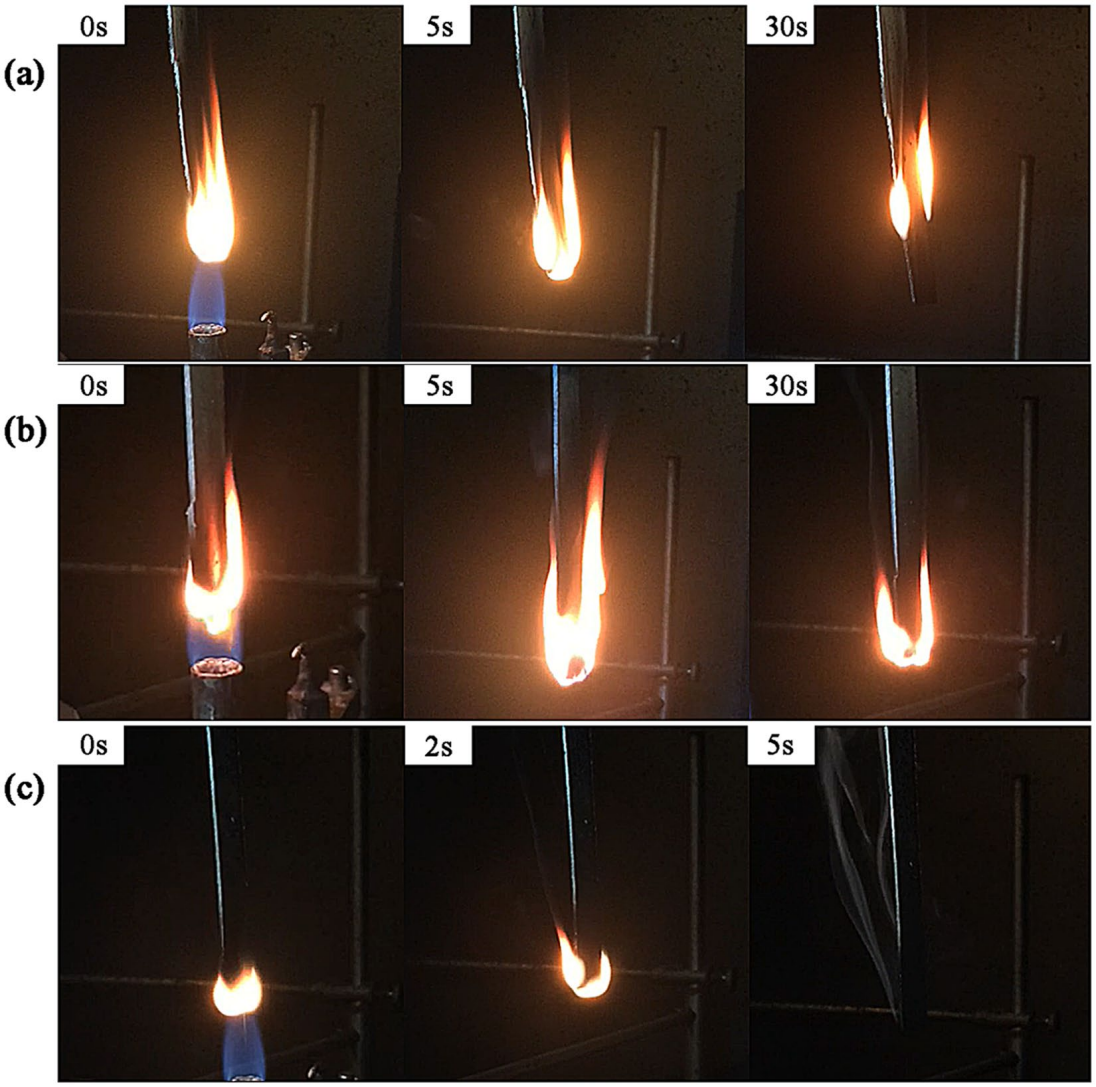
Vertical flame of the non-flame retardant EP composite (**a**), DPP/EP composite (**b**) and DPP-GO/EP composite (**c**).

The flame retardance of the different flame retardant EP composites was estimated by UL94 vertical burning test and LOI test (Table 2 and Fig. 8). The non-flame retardant EP composite had a very fast combustion rate without flame resistance. For DPP/EP composites, only a V-2 rating was achieved when the DPP content was increased to 8%. The vertical flame could not self-extinguish for GO/EP even with 8% GO content, indicating that GO alone had no obvious flame retardancy effect on the material. In contrast, the DPP-GO/EP composites exhibited greatly improved flame retardance, and the specimens could quickly self-extinguish after two ignitions, achieving a V-0 rating with only 4% DPP-GO content. In addition, after incorporating the flame retardants, the flame retardant EP composites showed increased LOI values compared with that of the neat EP resin. Moreover, a higher increase was obtained for the DPP-GO/EP composite than for the other flame retardant systems. The introduction of 4% DPP-GO increased the LOI value to 25.2%, further indicating the high flame retardant efficiency of DPP-GO for epoxy resin.

**Table 2:** UL94 and LOI values of the different flame retardant EP composites.

Samples	UL94 1.6 mm		LOI (%)
	t_1_/t_2_(s)	Rating	
Non-flame retardant EP composite	No self-extinction/-	No rating	19.8
4%DPP/EP composite	No self-extinction/-	No rating	22.6
8%DPP/EP composite	13.8/12.2	V2	23.8
2%GO/EP composite	No self-extinction/-	No rating	21.5
4%GO/EP composite	No self-extinction/-	No rating	22.0
8%GO/EP composite	No self-extinction/-	No rating	23.5
2%DPP-GO/EP composite	11.6/1.9	V1	23.6
4%DPP-GO/EP composite	3.0/3.2	V0	25.2

**Figure 9:**
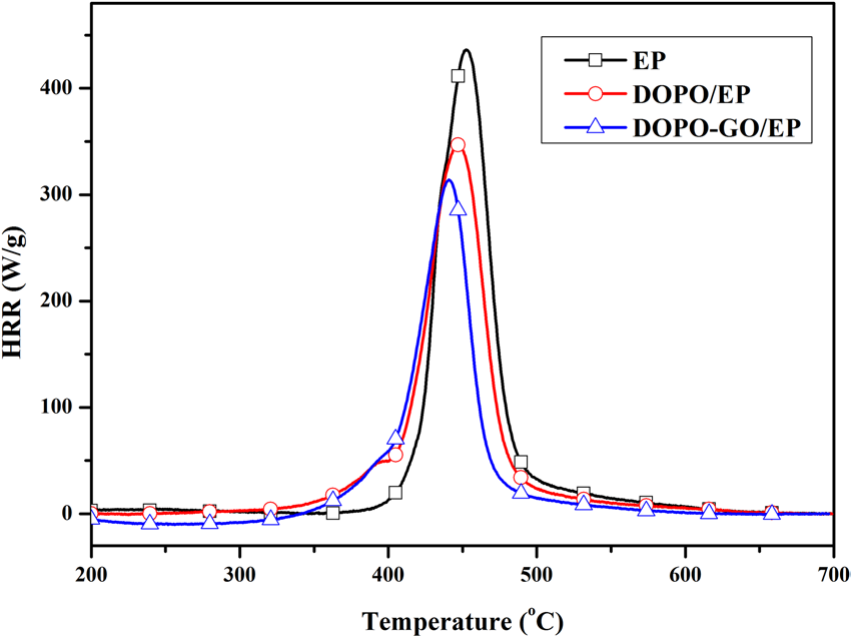
HRR curves of the non-flame retardant EP, DPP/EP and DPP-GO/EP.

MCC was adopted to further evaluate the heat release behavior of the flame retardant EP systems (without glass fiber). The plotted heat release rate (HRR) curves are shown in Fig. 9, and the related data were summarized in Table 3. The main heat release peak (PHRR) was recorded at about 450 °C in all three different systems. As the non-flame retardant EP degraded rapidly, it exhibited the highest PHRR and total heat release (THR) values. The DPP-GO/EP system showed remarkably decreased PHRR and THR values (30.8% and 35.6% reduction respectively) compared to non-flame retardant EP, but DPP/EP had only 20.5% and 7.8% reduction. These results indicated much lower fuel consumption rate (reflected by the oxygen consumption rate of the apparatus) of DPP-GO/EP compared to DPP/EP during combustion.

**Table 3:** Related data MCC of the flame retardant EP composites.

Samples	PHRR(W/g)	THR(kJ/g)
non-flame retardant EP	436.2	20.5
4%DPP/EP	346.8	18.9
4%DPP-GO/EP	301.9	13.2

The efficient flame retardance of DPP-GO relative to DPP can be explained as follows. As is well known, the mechanism of PFRs including DPP mainly relies on chemical production of a protective char layer that covers the material surface to isolate the fire. However, the production of the chemical char results from a catalysis reaction in the presence of phosphorus-containing acids at high temperature, accordingly, a certain amount of time is needed for the release of acids, as well as the formation of the barrier chars when flame occurs. This means that PFRs play ineffective roles during the initial combustion of materials. In comparison, DPP-GO itself is a carbon-based flame retardant, and its two-dimensional graphene sheets can provide barrier shields in the beginning of flame. Such an advantage overcomes the lagging effect of chemical charring to a degree. The following analysis further confirms the above mechanisms.

**Figure 10:**
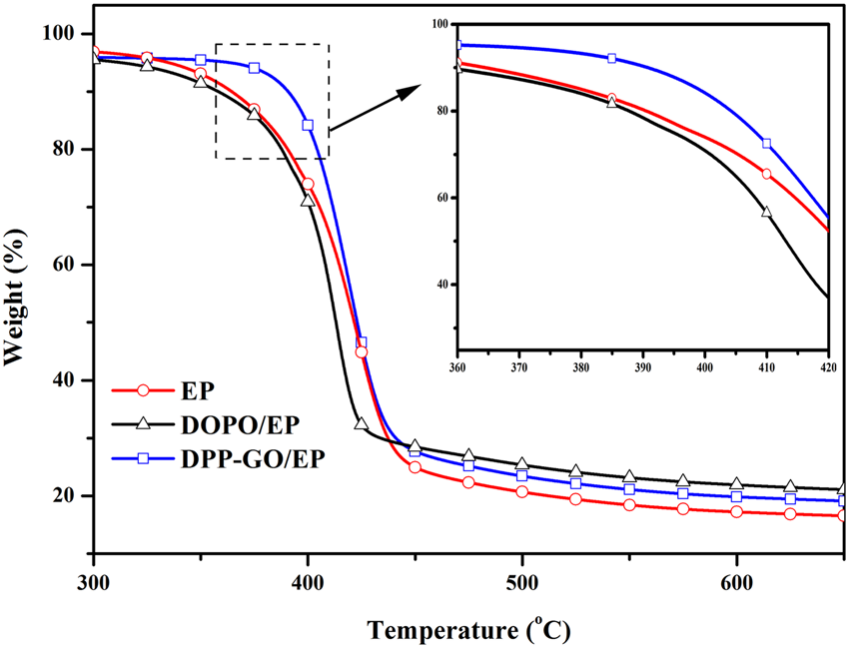
TGA curves of the non-flame retardant EP, DPP/EP and DPP-GO/EP.

The TG curves reflecting the influence of the additives on the thermal degradation of EP (without glass fibers) are shown in Fig. 10. In comparison with the non-flame retardant EP, the initial decomposition temperature of DPP/EP decreased at an earlier stage, showing that DPP accelerated the degradation of the resin due to the catalytic effect of the acid released by DPP. For DPP-GO/EP, its weight loss behavior occurred at higher temperature, demonstrating that combining DPP and GO enhances the thermal stability of the material. This result should be due to the two barrier effects of the materials: the graphene sheets effectively prevent the release of volatiles in the initial stage and, as a result, provide more time for later chemical charring behaviours by the interaction between DPP and the resin.

**Figure 11:**
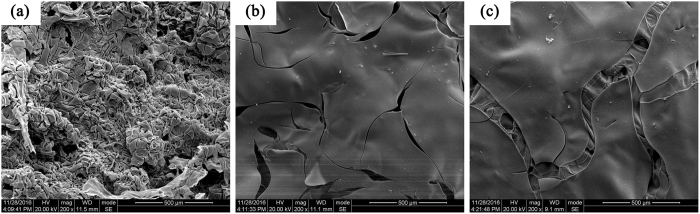
The residues morphologies of the non-flame retardant EP composite, DPP/EP composite and DPP-GO/EP composite after carbonization.

The different morphologies of the residues after carbonization are displayed in Fig. 11. The residues of the non-flame retardant EP showed a rough and loose structure. Introduction of DPP made the generated residue much more compact, but a number of cracks were observed on the surface of the chars. For the DPP-GO/EP system, the char layer was similarly smooth and some cracks were observed. However, these cracks seemed different from those of DPP/EP. Linkage structures were located in between the cracks to overcome the lack of the barrier. By contrast, there was nothing present in the cracks in the DPP/EP system, which led to a poorer barrier effect.

**Figure 12:**
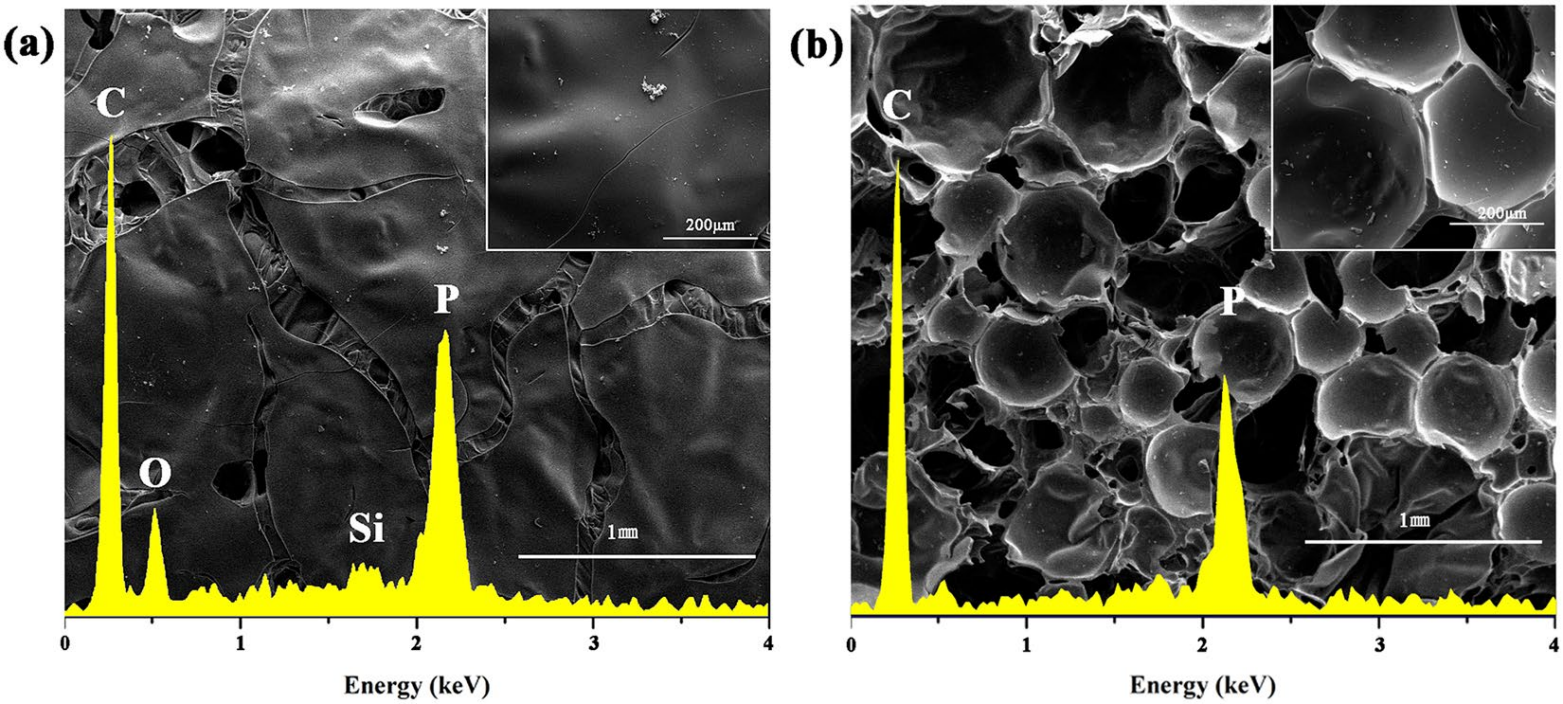
SEM images of the outer (**a**) and inner (**b**) layer of the DPP-GO/EP composite residues.

**Figure 13:**
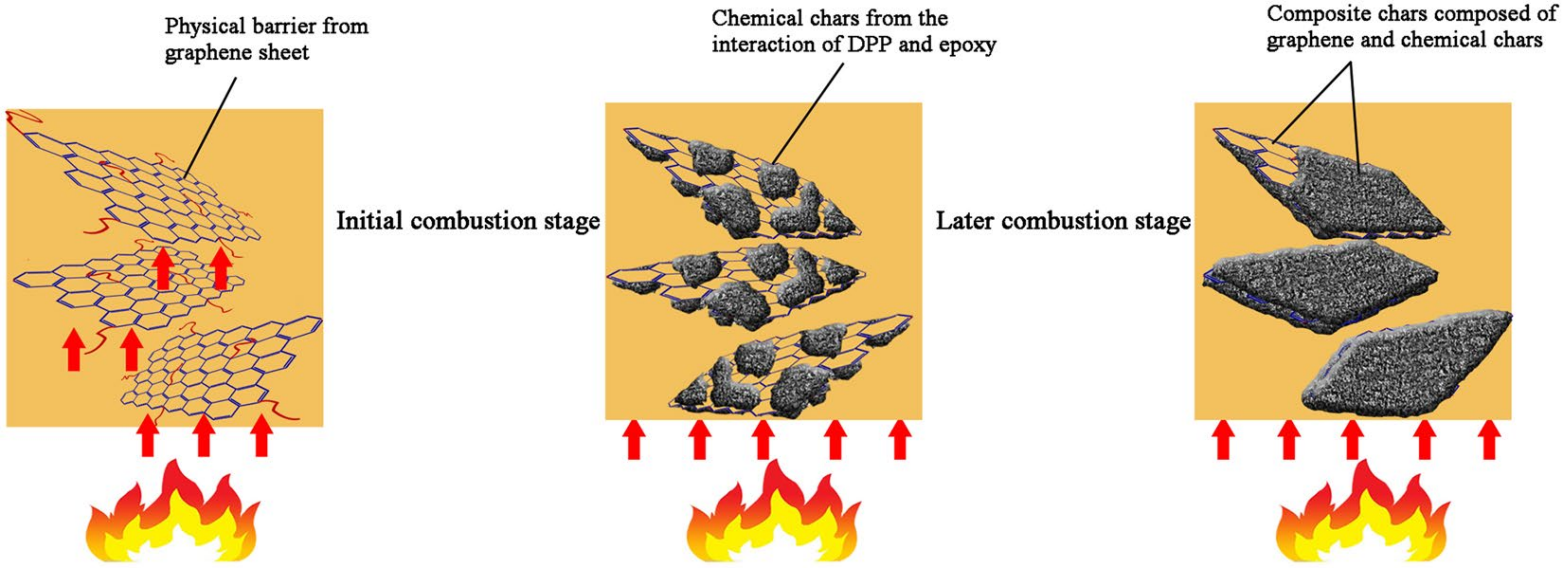
The flame retardant mechanism of the DPP-GO/EP system.

Additionally, it was found in Fig. 12 that the inner and outer residues of the DPP-GO/EP system showed totally different structures (no difference between the inner and outer residues of DPP/EP was observed). Combing the results of EDS analysis, the inner part was mainly composed of C and P element and, had a rough structure covered with many closed holes. Comparably, the outer layer possessed a smooth and lustrous surface, and its main elements were C, O, P and Si. According to the analysis, it is concluded that the two different char structures were generated by different processes: the inner layer is a physical char mainly consisting of graphene sheets, while DPP catalyzes the resin into chemical chars in the late stage to encapsulate the physical chars, thus constituting the outer layer. As a result, the combination of the two different chars can provide a very effective barrier in the condensed phase. The double barrier mechanism of the DPP-GO flame retardant system is described in Fig. 13.

### Mechanical properties of the DPP-GO flame retardant EP composites

**Figure 14:**
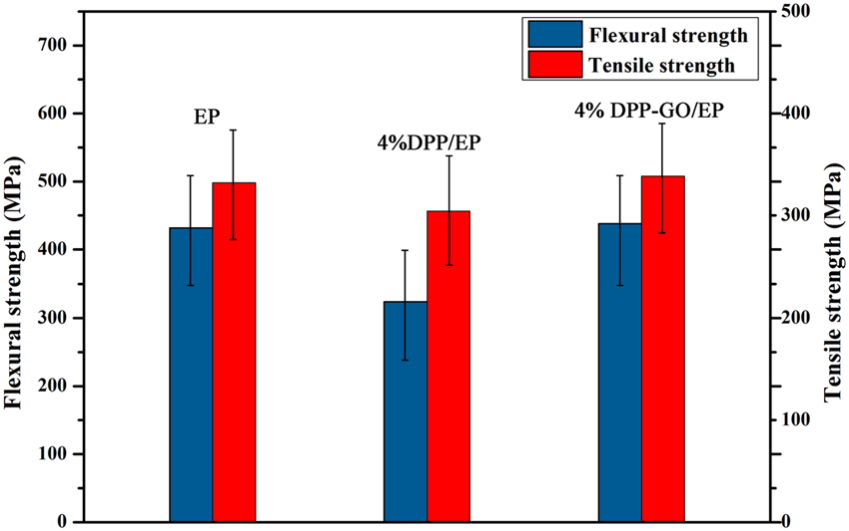
Mechanical properties the flame retardant EP composite laminates.

Finally, the mechanical properties of the flame retardant materials were evaluated (Fig. 14). It can be seen that the flexural strength and tensile strength of the DPP/EP laminate decreased by 24.9% and 32.1%, relative to the values of the non-flame retardant EP. However, for the DPP-GO/EP composite with the same additive content, the mechanical properties were slightly enhanced compared with those of EP. This indicated that the well dispersed graphene sheets reinforced the resin matrix to a degree. Evidently, this is an outstanding advantage of the DPP-GO flame retardant compared with conventional flame retardants which generally cause serious deterioration of the mechanical performance.

In summary, a graphene-based flame retardant was designed and successfully prepared by grafting long-chain phosphaphenanthrene, DPP, onto the GO surface. This system displayed promising results as a flame retardant EP laminate. The hybrid flame retardant had the following advantages, leading to potential commercial prospect.

Double barrier effects: the GO sheets effectively blocked and delayed the rapid release of volatile gases during the initial combustion stage, and afterwards, composite chars (chemical chars encapsulating the GO char) were formed to greatly improve the barrier properties in the condensed phase.

High exfoliation and dispersion: the long-chain and bulky group grafted onto the GO sheets, weakened the van der Waals forces and expanded the space between neighboring graphene sheets due to steric hindrance, which is advantageous for obtaining a functional resin with well-dispersed flame retardant.

## Methods

### Materials

Graphite powder was kindly supplied by Nanjing XFNano Materials Tech Co., Ltd. Potassium permanganate (KMnO_4_), sulfuric acid (H_2_SO_4_, 98%), hydrogen peroxide (H_2_O_2_), sodium nitrate (NaNO_3_), N,N-dimethylformamide (DMF), dimethyl sulfoxide (DMSO), thionyl chloride (SOCl_2_), tetrahydrofuran (THF), ethanol and acetone were purchased from Aladdin Chemical Co., Ltd. 9,10-dihydro-9-oxa-10-phos-phaphenanthrene-10-oxide (DOPO) and Silane coupling agent (SCA), g-glycidyloxypropyltrimethoxysilane, was purchased from Chengdu Thinkbond Chemical Co. Ltd., China. Epoxy resin (diglycidyl ether of bisphenol-A type, epoxy equivalent weight: 489 g per eq.) was provided by Huntsman Advanced Materials Co., Ltd., and a phenolic novolac resin hardener (hydroxyl equivalent weight: 105 g per eq.) was supplied by Momentive Chemical Co., Ltd. Glass fabrics, 7628#, were obtained from Jushi Group Co., Ltd. DPP was synthesized in our laboratory.

### Synthesis of DPP-GO

**Figure 15:**
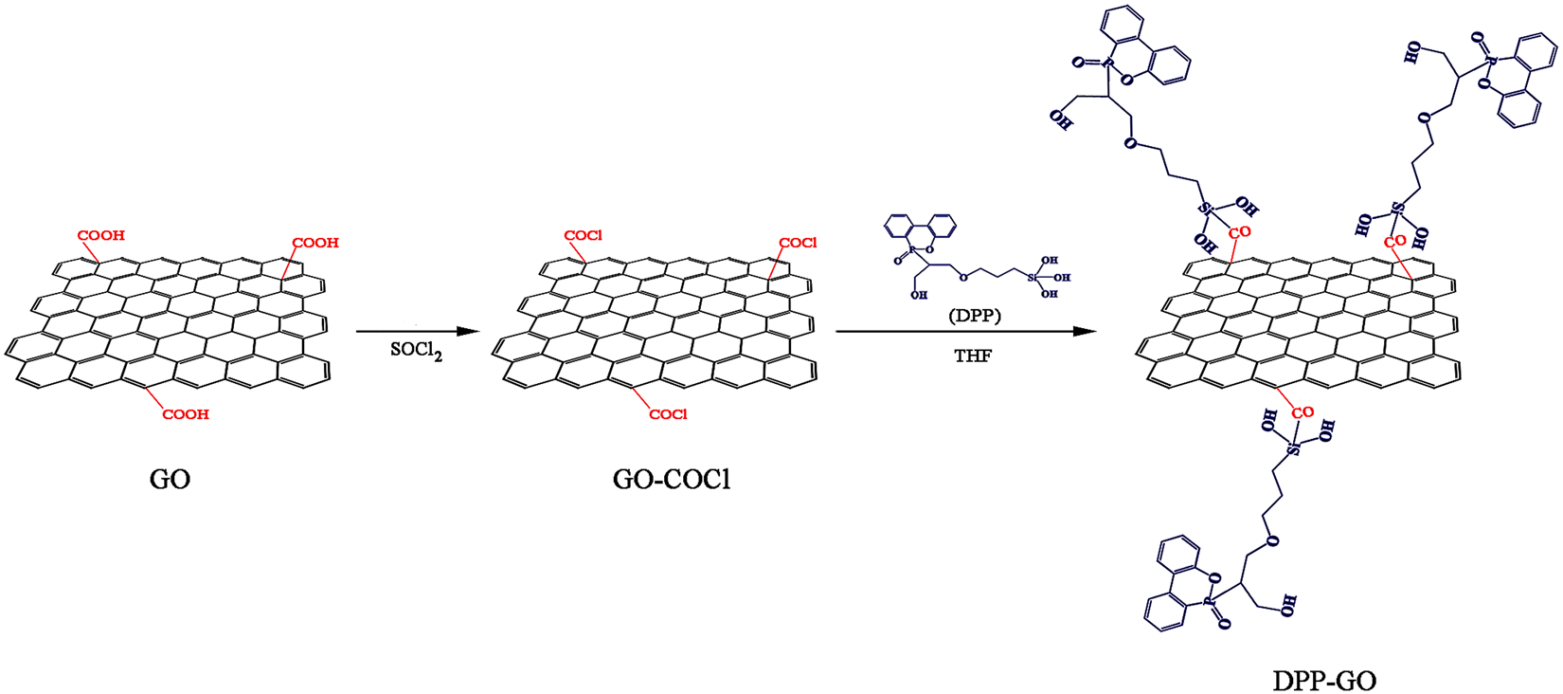
Synthetic route to obtain the graphene-based hybrid flame retardant.

First, GO was prepared from graphite using the modified Hummers method^28, 29^. A mixture of graphite (2.5 g) and NaNO_3_ (2.5 g) was diluted in 120 ml of concentrated H_2_SO_4_, and the mixed solution was cooled to 0 °C in an ice bath. KMnO_4_ (15 g) was added slowly in small doses to maintain the reaction temperature below 20 °C. Then, the solution was heated to 50 °C and stirred for 12 h. Then H_2_O_2_ was added slowly with stirring for 30 min, and finally, the mixture was centrifuged. The remained solid material was then washed with water and centrifuged again until the pH was neutral. Then, the obtained GO from the acid reaction (200 mg) was refluxed in 40 ml of SOCl_2_ in the presence of 1.0 ml of DMF at 70 °C for 24 h, using a CaCl_2_ guard tube. The excess SOCl_2_ was removed by distillation after the end of the reaction. The modified GO (0.5 g) dispersed in THF (100 ml) was added into a 250 ml three-necked flask equipped with a mechanical stirrer, nitrogen inlet and reflux condenser. A calculated amount of DOPO powder and SCA were mixed in an oven and heated for 6 h at 160 °C, then the obtained mixture was dissolved in ethyl alcohol–water (weight ratio = 1: 2) mixed solvent with stirring for 30 min at room temperature. The DPP was prepared after the excess solvent was removed by distillation. DPP (2.0 g) was added to the suspension with stirring, and the reaction was performed at 70 °C for 24 h. The obtained DPP-GO was filtered, washed with THF and ethanol, and then dried under vacuum at 60 °C for 24 h. Figure 15 shows the synthesis of DPP-GO.

### Preparation of the flame retardant EP

**Figure 16:**
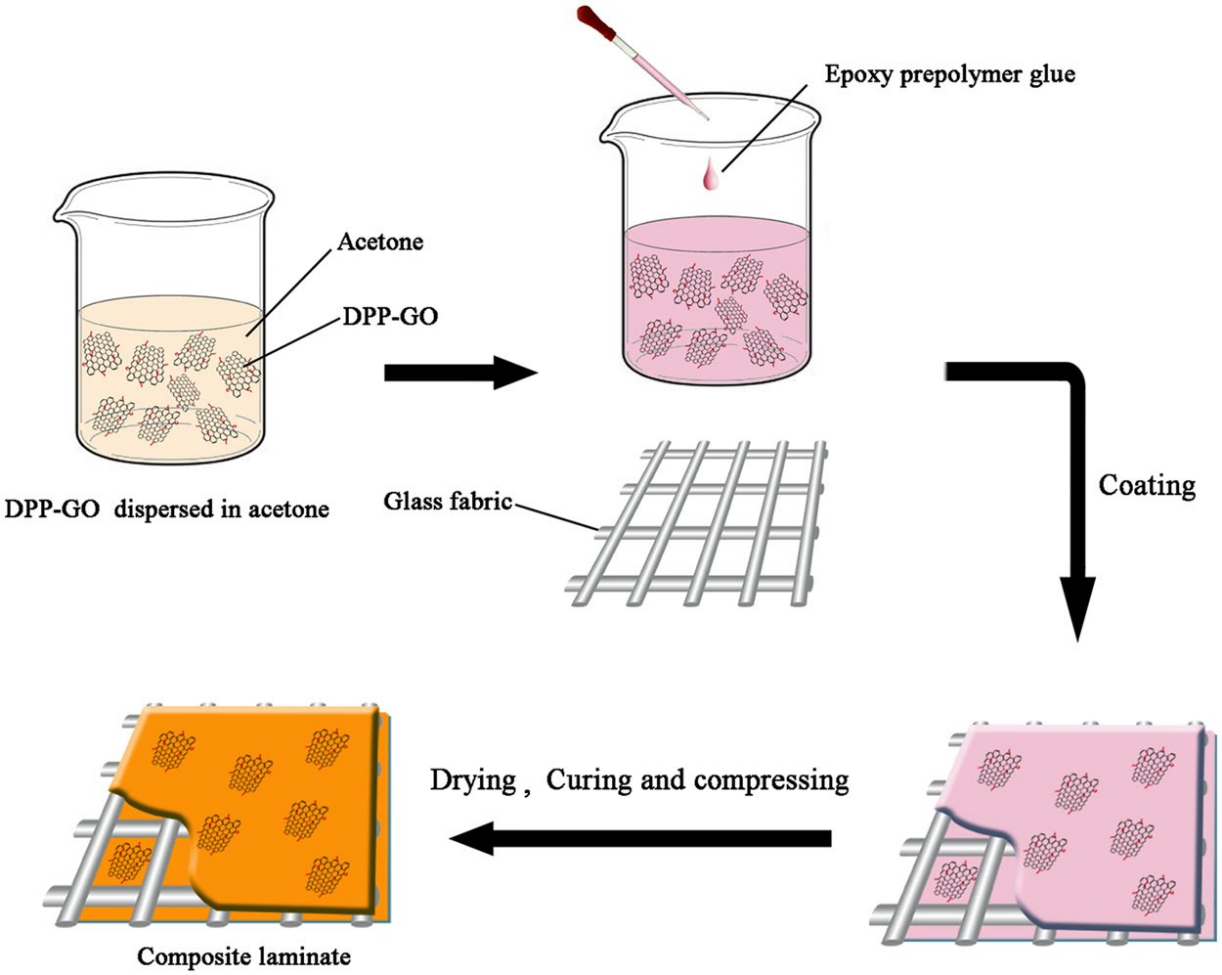
Preparation process of the flame retardant EP laminate composites.

The flame retardant (DPP or DPP-GO) was first suspended in acetone and sonicated for 10 min in an ultrasonic bath. Subsequently, the EP prepolymer glue (including 7.50 g resin and 1.7 g phenolic novolac resin) was added. Then the above flame retardant EP glue was stirred for 30 min and evenly coated on a piece of glass fabric, which was then heated in an oven at 155 °C for 5 min to remove the solvent. Subsequently, a certain number of glass fabric pieces (the number was adjusted by the thickness of the test samples: 8 pieces for 1.6 ± 0.1 mm and 10 pieces for 2.0 ± 0.1 mm) were laminated and cured in a vulcanizing machine through a heating program at 190 °C for 2 h. Finally, the obtained laminates were cut into the standard bars. The corresponding preparation procedure is illustrated in Fig. 16.

### Characterization

The Fourier transform infrared (FT-IR) spectra were recorded on a Nicolet 20SXB infrared spectrometer (Thermo Fisher USA). X-ray photoelectron spectroscopy (XPS) was conducted using a Shimadzu/Kratos AXIS Ultra DLD multifunctional X-ray photoelectron spectrometer (Manchester, UK). The morphology and structure of GO and DPP-GO were studied by transmission electron microscopy (TEM) using a Tecnai G2 F20 electron microscope at an accelerating voltage of 200 kV. TG analysis was performed using a TA Q-500 TGA thermal analyzer at a heating rate of 10 °C /min, over the temperature range of 30 to 650 °C, with a nitrogen flow of 100 ml/min. Approximately 8~10 mg of the sample was used in each test. XRD patterns were recorded using a DX-1000 diffractometer (Dandong Fangyuan Instrument Co., Ltd, China), with a CuKα generator system operated at 40 kV and 25 mA, over a 2θ range of 5° to 40° at a scanning rate of 1°/s. Raman spectra were recorded on a Labram HR spectrometer (HORIBA Jobin Yvon) using 532 nm laser excitation with a power of 1 mW. The vertical burning tests were conducted on a HK-HVR vertical burning tester (Zhuhai Huake Testing Equipment Co., Ltd) with the dimensions of 127 × 12.7 × 1.6 mm^3^ according to the American National UL-94 test (ANSI/UL 94-2013). The LOI was measured using an automatic oxygen index analyzer (Shandong Textile Science Research Institute) according to ASTM D2863. The sample dimensions were 120 × 6.5 × 3.0 mm^3^. Microscale combustion calorimetry analysis was carried out by using a FAA-PCFC microscale combustion calorimeter (Fire Testing Technology Limited UK), and about 2 mg powder (without glass fiber involved) scraped from the EP composites surface was heated from the ambient temperature to 800 °C at a heating rate of 1 °C/s under air atmosphere. The surface morphology and elemental composition of the samples carbonized at 600 °C for 10 min were observed by using a scanning electronic microscope (SEM) (JSM-5900LV, JEOL Ltd., Tokyo, Japan) with a conductive gold layer coating at an accelerating voltage of 10 kV. The mechanical properties, including tensile strength and flexural strength, of the composites were measured at ambient temperature using a RGM-4010 university testing machine of (ShenZhen Reger Instrument Co. Ltd, China) according to ASTM D638-10 and ASTM D790-10, respectively.
